# Probable PTSD, depression and anxiety in 40,299 UK police officers and staff: Prevalence, risk factors and associations with blood pressure

**DOI:** 10.1371/journal.pone.0240902

**Published:** 2020-11-12

**Authors:** Sharon A. M. Stevelink, Elena Opie, David Pernet, He Gao, Paul Elliott, Simon Wessely, Nicola T. Fear, Matthew Hotopf, Neil Greenberg

**Affiliations:** 1 King’s Centre for Military Health Research, Department of Psychological Medicine, King’s College London, London, United Kingdom; 2 Department of Psychological Medicine, King’s College London, London, United Kingdom; 3 MRC Centre for Environment and Health, School of Public Health, Faculty of Medicine, Imperial College London, London, United Kingdom; 4 NIHR Imperial College Biomedical Research Centre, Imperial College London, London, United Kingdom; 5 UK Dementia Research Institute at Imperial College London, Hammersmith Hospital Campus, London, United Kingdom; 6 Health Data Research UK London at Imperial College London, Faculty of Medicine, St Mary’s Campus, London, United Kingdom; 7 Academic Department of Military Mental Health, King’s College London, London, United Kingdom; 8 South London and Maudsley NHS Foundation Trust, London, United Kingdom; University of the Witwatersrand, SOUTH AFRICA

## Abstract

**Introduction:**

Police employees undertake challenging duties which may adversely impact their health. This study explored the prevalence of and risk factors for probable mental disorders amongst a representative sample of UK police employees. The association between mental illness and alterations in blood pressure was also explored.

**Methods:**

Data were used from the Airwave Health Monitoring Study which was established to monitor the possible physical health impacts of a new communication system on police employees. Data included sociodemographic characteristics, lifestyle habits, depression, anxiety, and post-traumatic stress disorder (PTSD) symptoms and blood pressure. Descriptive statistics were used to explore the prevalence of probable mental disorders and associated factors. Stepwise linear regression was conducted, controlling for confounding variables, to examine associations between mental disorders and blood pressure.

**Results:**

The sample included 40,299 police staff, police constable/sergeants and inspectors or above. Probable depression was most frequently reported (9.8%), followed by anxiety (8.5%) and PTSD (3.9%). Groups at risk for probable mental disorders included police staff, and police employees who reported drinking heavily. Police employees exposed to traumatic incidents in the past six months had a doubling in rates of anxiety or depression and a six-fold increase in PTSD compared to those with no recent trauma exposure. Adjusted logistic regression models did not reveal any significant association between probable mental disorders and systolic blood pressure but significantly elevated diastolic blood pressure (≈1mmHg) was found across mental disorders.

**Conclusions:**

These results show lower rates of probable mental disorders, especially PTSD, than reported in other studies focusing on police employees. Although mental ill health was associated with increased diastolic blood pressure, this was unlikely to be clinically significant. These findings highlight the importance of continued health monitoring of members of the UK police forces, focusing on employees recently exposed to traumatic incidents, heavy drinkers and police staff.

## Introduction

Across England, Wales and Scotland, the number of police officers amounts to over 140,000 individuals [1]. It is commonly assumed that policing is a particularly stressful occupation with elevated rates of mental disorders, and that this is largely due to the high rates of exposure to traumatic events [2–4]. A study published in 2003 investigating the mental health of UK police officers identified that 41% reported symptoms of common mental disorders (CMDs) such as depression and anxiety [5]. In other Western countries, findings revealed that 25% of police officers met criteria indicative of problematic drinking, 22% reported symptoms of PTSD [6], and 16% met the criteria for depression [7]. Other work shows that while traumatic events are certainly part of the life of operational police employees, they are not necessarily the sole, or even most important source of stress [5,8]. In fact, studies have shown that some of the most impactful causes of stress are occupational in nature, such as lack of control over work load, job routines, impact of work demands on family life and inadequate support [5,8–10]. Whereas these occupational stressors may not frequently cause mental disorders directly, they may contribute to elevated levels of stress and making police employees more vulnerable to the development of mental disorders when they are exposed to traumatic events [7]. Indeed, routine work stressors were found to be more strongly associated with PTSD symptoms than traumatic events experienced in the line of duty among US police officers [11].

There are limitations with estimating mental disorder prevalence derived from occupational studies instead of population studies. Research has suggested that when studies select people based on being part of an occupational group or focus on exploring adverse mental health outcomes, higher prevalence estimates are found due to a contextual or response bias [12]. In this paper, we used data from a study which included a representative sample of UK police employees (*n* > 40,000) and was carried out with the primary purpose of investigating concerns associated with the introduction of a new communication system, namely the terrestrial trunked radio (TETRA), and its possible impacts on physical health. The opportunity was also taken to examine other issues, principally cardiovascular risk factors and genetic epidemiology [13]. We used this data to explore the prevalence of, and factors associated with, probable mental disorders (e.g. PTSD, anxiety and depression) among police employees. As the original study focused on physical health outcomes, instead of mental health outcomes, we would expect the impact of a response bias to be limited in our sample. Therefore, lower, and more accurate, prevalence estimates of probable mental disorders are expected in the current study, compared to studies framed as an occupational stress or mental health study.

A second area of interest for this paper, is the relationship between occupation, stress and cardiovascular events [14,15]. Psychological stressors have been identified as important risk factors for cardiovascular disease [16]. Cardiovascular reactivity in response to acute or prolonged stressors may result in the hyperactivity of the hypothalamic-pituitary-adrenal (HPA) axis as well as the sympathetic nervous system (SNS), leading to cardiovascular changes such as increased heart rate and blood pressure. The impact of psychological stressors may be compounded by the clustering of cardiovascular risk factors among those working in high stress occupations, such as the police, including high levels of obesity, smoking, drinking, a sedentary lifestyle, shift work and disrupted sleep patterns [17].

However, findings concerning the association between high blood pressure, work-related stress and mental disorders are inconsistent. For example, conflicting evidence is found among air traffic controllers, a stressful occupation due to the nature of the work, whereby no differences were found in cardiovascular outcomes such as blood pressure readings taking during the day, at night, over 24 hours or hourly compared to controls [18]. However, a longitudinal study conducted in air traffic controllers found that those who showed elevated systolic cardiovascular reactivity due to work stress at baseline, were more likely to be suffering from new-onset hypertension two decades later [19]. Another study found that people reporting PTSD symptoms had increased blood pressure compared to controls [20] whereas no significant positive association was found between increased blood pressure and symptoms of depression in a different study [21]. A study using data from the World Mental Health Surveys (*n =* 52,095) found a positive association between the first occurrence of common mental disorders, alcohol misuse and subsequent onset of hypertension [22]. Findings among emergency services personnel have shown that potentially life-threatening activities may produce adrenergic surges, which are associated with an increased risk of cardiovascular events [14,23], and that lack of control over workload can increase both systolic (SBP) and diastolic blood pressure (DBP) [24,25]. The limited research on police personnel, mostly done in the US, suggests that those with the highest PTSD symptoms scores were three times more likely to meet the criteria for metabolic syndrome (a clustering of cardiovascular disease risk factors i.e. high blood pressure, obesity) than those with lower PTSD scores [26].

Investigating the mental health and associated factors among members of the UK police force is important as mental ill health is a key cause of absenteeism and presenteeism and thus directly impacts on the country’s readiness to respond to emergencies. Failure to look after the police’s health and wellbeing may hamper their ability to protect the public and may result in safety risks to self and others whilst being on duty. However, to date, most research findings focusing on members of the UK police forces have been derived from studies with small sample sizes and police officers being included from a few select police departments, hence findings are not generalisable.

This study explored the prevalence of probable mental disorders namely PTSD, anxiety and depression, amongst a large representative sample of members of the UK police forces, associated risk factors and examined whether mental ill health is associated with alterations in blood pressure. We anticipate that these findings can be used to inform policies and interventions to maintain the mental health and wellbeing of the UK police forces.

## Materials and methods

### Sample and design

Cross-sectional data from the Airwave Health Monitoring Study, led by Imperial College London, were used. This is an occupational study launched in June 2004 that, by 2015, enrolled 53,114 police officers and staff (defined as ‘police employees’) from across Great Britain police forces. The design and rationale for the Airwave Health Monitoring Study (‘Airwave Study’ hereafter), have been described previously, but we will summarise the essentials below [13].

### Data collection

The Airwave Study was set up to investigate concerns about the possible health effects of TETRA for the police forces [13]. This digital radio system was introduced in 2001 and is used by other emergency services in the UK. Extensive data on police personnel were attained via the baseline survey that took place between 2004–2015. The study included two phases. In phase 1, all police employees received an enrolment questionnaire via various channels as deemed appropriate by the participating police forces. The enrolment questionnaire was made available electronically and via paper. Then in phase 2, a health screen was performed by trained nurses. Respondents were recruited via publicity of a full, free health screen as well as participation in a major research project on the health of police employees. Together, this baseline survey covered demographics, work environment (e.g. working hours per week, overtime), TETRA usage (e.g. type of radio, patterns of usage) as well as lifestyle, health and wellbeing assessments including medical history, 7-day food diaries, cognitive testing, blood and urine samples and a variety of physical measurements including standing and sitting height, pulse rate and blood pressure. The questionnaire booklet also included some measures on mental health namely the Trauma Screen Questionnaire (TSQ), the Patient Health Questionnaire (PHQ) and the Hospital Anxiety and Depression Scale (HADS-A) [13,27–29].

### Measures

The main outcomes of interest in this paper were probable mental disorders, and subsequently its relationship with blood pressure. Probable mental disorders included depression, anxiety, and PTSD. Probable depression was measured using the 9-item PHQ-9. Response options ranged from ‘not at all’ (0) to ‘nearly every day’ (3) and a score of ≥10 was used as a cut off indicative of probable depression [30,31]. Probable anxiety was measured using the anxiety subscale of the HADS-A. Response options ranged from ‘not at all’ (0) to ‘most of the time’ (3) and a score of >10 was used as a cut off indicative of probable anxiety [32]. Probable PTSD was measured using the TSQ. Response options ranged from ‘not at all’ to ‘extremely’. Any response other than ‘not at all’ was scored as 1 and a score of ≥6 was used as a cut off indicative of probable PTSD [33]. The TSQ was only asked to respondents who said ‘yes’ to the following screening question: Have you been bothered by a disturbing incident which has occurred over the past 6 months? All mental health measures have been widely used and have shown to be valid and reliable across samples [30,32–35]. The reliability of the PHQ, HADS-A and TSQ in the Airwave sample was good, α = 0.84, α = 0,83 and α = 0.93 respectively. Pulse rate and blood pressure were measured by a trained using a sphygmomanometer when participants reported for the health screen at phase 2. Three separate readings were taken consecutively, each of which recorded systolic [SBP mmHg] and diastolic blood pressure [DBP mmHg]. Blood pressure was taken in a seated position, unless a participant self-reported being diabetic. In this case, two sets of readings were taken, one standing and one sitting. Subsequently, the mean was calculated from all measurements taken. Responders were also asked whether they were diagnosed by a clinician with hypertension.

Other variables included in this paper were sociodemographic characteristics including gender, age, marital status, ethnicity, educational attainment, exercise (e.g. ‘do you undertake intense exercise’), smoking, and BMI (measured through direct clinical evaluation). Police role was based on self-report by the respondents (e.g. police staff, police constable/sergeant, inspector or above). In general, it is assumed that police staff would not routinely respond to incidents directly and carry out support roles and thus are not likely to be directly exposed to traumatic events. However, they may work with traumatic materials and with officers (e.g. constables/sergeants, inspectors etc) who are responding to such events and have a more frontline role. Alcohol consumption was based on respondents reporting their alcohol consumption of beer, wine and spirits over the past 7-days. We classed respondents in four groups, based on the latest UK Chief Medical’s Officers recommended guidelines for weekly units. Non-drinkers included those who said ‘no’ to the following question ‘do you currently drink alcohol’, ‘low risk’ included those drinking less than 14 units for both men and women, ‘increasing risk’ 14–35 units for women and 14–50 units for men, ‘high risk’ above 35 units for women and above 50 units for men [36,37].

### Data analysis

The data were analysed using the statistical package STATA v.15 (StataCorp. College Station, TX, USA). Data on blood pressure and mental health status were available for 41,038 respondents out of the 53,114 [38]. Not all participants attended the health screening during which the anthropometric and bioassay results were collected or completed an online questionnaire that included additional measures, including mental health. Further, as the mental health questions were only introduced at a later stage, some individuals were missed. Individuals without this information were dropped from the sample that we obtained (n = 12,076). We dropped an additional 739 respondents as their role was classified as ‘other’ as they were not necessarily part of the active police force at the time or they did not consider themselves as inspectors, police constable/sergeants or police staff. The final sample was 40,299 police employees.

Descriptive statistics were used to explore mental health outcomes across different sociodemographic, lifestyle and job characteristics (*i*.*e*. Chi squared test for categorical variables, t-test for the comparison of two means for continuous variables). A more detailed descriptive analysis was done exploring mental health outcomes among police personnel who reported exposure to a traumatic event in the past six months. Linear stepwise regression models were run to examine the association between mental illness and blood pressure. Multivariate linear regression models adjusted for the following confounders were conducted as informed by the literature [2,14,20,39]: gender, age, educational attainment, marital status, ethnicity, job role, BMI, exercise, smoking and alcohol consumption. Missing data were limited across the variables of interest (<10%) and hence participants were dropped if they had no data on the outcome of interest for specific analyses.

### Ethics

The Airwave Health Monitoring Study received ethical approval from the National Health Service multi-site research ethics committee (MREC/13/NW/0588). Written informed consent was obtained from all participants.

## Results

The mean age of the 40,299 police employees was 40.8 (SD 8.9) and 63.0% were male. The majority were married or cohabiting (77.7%) and were white (94.8%). Most were police constables and sergeants (63.8%) followed by police staff (28.6%) and inspectors (7.6%). Less than 10% were smokers and 9.1% of police employees were not drinking alcohol. Most police employees were low risk drinkers (55.2%) followed by increased risk (32.6%) and a small proportion were drinking heavily (3.0%) (Table 1).

**Table 1:** Characteristics of police employees across probable mental disorders.

Characteristics	All participants	Probable depression (PHQ ≥10)			Probable anxiety (HADSA ≥11)			Probable PTSD (TSQ ≥6)		
		No	Yes	p-value	No	Yes	p-value	No	Yes	p-value
**Overall** (n (%)	40299 (100.0)	35708 (90.2)	3870 (9.8)		36229 (91.5)	3349 (8.5)		36318 (96.1)	1474 (3.9)	
**Gender** n (%)				<0.001			<0.001			0.211
Female	14900 (37.0)	12835 (87.5)	1827 (12.5)		12859 (87.7)	1803 (12.3)		13543 (96.3)	526 (3.7)	
Male	25399 (63.0)	22873 (91.8)	2043 (8.2)		23370 (93.8)	1546 (6.2)		22775 (96.0)	948 (4.0)	
**Age** n (%)				<0.001			0.045			<0.001
< 30 years	5278 (13.1)	4604 (88.8)	580 (11.2)		4717 (91.0)	467 (9.0)		4772 (96.7)	163 (3.3)	
30–39 years	13177 (32.7)	11648 (89.9)	1305 (10.1)		11859 (91.6)	1094 (8.5)		11869 (96.3)	460 (3.7)	
40–49 years	15929 (39.5)	14132 (90.4)	1493 (9.6)		14279 (91.4)	1346 (8.6)		14237 (95.5)	668 (4.5)	
> 49 years	5915 (14.7)	5324 (91.5)	492 (8.5)		5374 (92.4)	442 (7.6)		5440 (96.8)	183 (3.3)	
**Marital status** n (%)				<0.001			<0.001			0.337
Married/cohabiting	31127 (77.7)	28087 (91.5)	2624 (8.5)		28331 (92.3)	2380 (7.8)		28173 (96.2)	1126 (3.8)	
Single	4758 (11.9)	4056 (86.1)	653 (13.9)		4207 (89.3)	502 (10.7)		4326 (96.2)	173 (3.9)	
Divorced/separated	3239 (8.1)	2722 (85.1)	475 (14.9)		2830 (88.5)	367 (11.5)		2931 (95.9)	127 (4.2)	
Other	918 (2.3)	802 (87.8)	111 (12.2)		815 (89.3)	98 (10.7)		847 (95.1)	44 (4.9)	
**Ethnicity** n (%)				<0.001			0.704			0.216
White	37926 (94.8)	33787 (90.4)	3605 (9.6)		34226 (91.5)	3166 (8.5)		34325 (96.1)	1380 (3.9)	
Other	2087 (5.2)	1816 (87.9)	250 (12.1)		1896 (91.8)	170 (8.2)		1882 (95.6)	87 (4.4)	
**Education** n (%)				<0.001			0.055			0.491
Vocational qualifications	2818 (7.0)	2464 (88.6)	318 (11.4)		2553 (91.8)	229 (8.2)		2545 (95.6)	116 (4.4)	
GCSE equivalent or below	13374 (33.4)	11803 (89.6)	1375 (10.4)		12016 (91.2)	1162 (8.8)		11963 (96.1)	486 (3.9)	
A levels/higher or equivalent	12728 (31.8)	11369 (90.4)	1210 (9.6)		11580 (92.1)	999 (7.9)		11606 (96.1)	475 (3.9)	
Bachelor/postgraduate degree	11122 (27.8)	10031 (90.2)	960 (8.7)		10034 (91.3)	957 (8.7)		10163 (96.3)	393 (3.7)	
**Role** n (%)				<0.001			<0.001			<0.001
Police staff	10406 (28.6)	9082 (88.9)	1134 (11.1)		9156 (89.6)	1060 (10.4)		9445 (97.0)	289 (3.0)	
Police constable/sergeant	23256 (63.8)	20606 (90.4)	2181 (9.6)		21009 (92.2)	1778 (7.8)		20688 (95.7)	931 (4.3)	
Inspector or above	2782 (7.6)	2569 (93.9)	166 (6.1)		2555 (93.4)	180 (6.6)		2487 (95.7)	112 (4.3)	
**Takes intensive exercise** n (%)				<0.001			<0.001			0.831
No	35670 (88.8)	31472 (89.9)	3556 (10.2)		31962 (91.3)	3066 (8.8)		32136 (96.1)	1304 (3.9)	
Yes	4508 (11.2)	4130 (93.3)	299 (6.8)		4158 (93.9)	271 (6.1)		4067 (96.0)	168 (4.0)	
**Currently smoking** n (%)				<0.001			<0.001			0.857
No	36286 (90.2)	32323 (90.7)	3317 (9.3)		32720 (91.8)	2920 (8.2)		32729 (96.1)	1328 (3.9)	
Yes	3961 (9.8)	3340 (85.9)	547 (14.1)		3463 (89.1)	424 (10.9)		3541 (96.0)	146 (4.0)	
**Alcohol consumption#** n (%)				<0.001			<0.001			<0.001
Non-drinker	3649 (9.1)	3151 (87.4)	455 (12.6)		3234 (89.7)	372 (10.3)		3323 (95.7)	149 (4.3)	
Low risk	22097 (55.2)	19754 (90.6)	2047 (9.4)		20082 (92.1)	1719 (7.9)		20240 (96.6)	706 (3.4)	
Increasing risk	13061 (32.6)	11683 (91.1)	1136 (8.9)		11746 (91.6)	1073 (8.4)		11565 (95.6)	538 (4.5)	
High risk	1211 (3.0)	968 (81.4)	221 (18.6)		1019 (85.7)	170 (14.3)		1027 (92.8)	80 (7.2)	
**BMI** (kg/m2)	27.22 (4.2)	27.10 (4.1)	28.32 (5.0)	<0.001	27.20 (4.2)	27.37 (4.7)	0.025	27.15 (4.2)	28.24 (4.6)	<0.001

BMI, body mass index; GCSE, General Certificate of Secondary Education; HADSA, hospital anxiety and depression scale; PHQ, patient health questionnaire; PTSD, post-traumatic stress disorder; SD, standard deviation; TSQ, trauma screening questionnaire. ^#^Alcohol drinking was based on alcohol units calculated from different type of drinks/beverages consumed in the past week using sex-specific cut offs: non-drinker, not drinking alcohol; low risk <14 units men and women; increasing risk 14–35 units for women, 14–50 units for men; high risk >35 units women and >50 units for men. was missing if a participant did not attend the health screening. Other missing values were due to no assessment of those participant characteristics in a specific version of the questionnaire or no response received from participants. P-values have been derived from the Chi2 test for categorical variables and independent t-test for continuous variables.

### Mental health

The prevalence of probable mental disorders varied, with probable depression being most frequently reported in 9.8% (95% CI 9.5–10.1) of police employees, probable anxiety in 8.5% (95% CI 8.2–8.7) and, probable PTSD in 3.9% (95% CI 3.7–4.1) (Table 1). Reporting more than one probable mental disorder occurred in 5.6% of respondents. As expected, lower ranked employees (constable/sergeant and staff) were the most at risk for probable mental disorders across conditions compared to higher ranks (inspector or chief inspector, superintendent and above).

Similar risk factors were identified for reporting symptoms of anxiety and depression. Women were significantly more likely to be affected, as well as those of younger age, not in a relationship, and lower educational attainment. Police employees with less healthy lifestyle habits such as smoking, high risk drinkers and those who were less likely to exercise reported higher levels of probable anxiety and depression. Less variation was seen regarding the prevalence of probable PTSD. We identified that police employees aged 40–49 years reported the highest levels of probable PTSD (4.5%) and high-risk drinkers (7.2%).

A different picture emerged when only including police officers and staff (*n* = 5,454 (14.4%)) who reported exposure to a traumatic experience in the last six months (S1 Table). In this group, the prevalence of probable depression and anxiety approximately doubled to 17.8% and 16.4% respectively. Women, those not in a relationship, police staff and those with less healthy lifestyle habits (e.g. smoking, heavy alcohol consumption) reported the highest rates of anxiety and depression. Further, the highest prevalence was found in police officers and staff who reported to be drinking heavily, with 38.7% meeting criteria for depression and 32.0% meeting criteria for anxiety. Twenty-seven percent of recently trauma exposed employees met criteria for probable PTSD, with police staff (31.8%) and those consuming alcohol at an increasing risk to health (29.8%) and high risk to health (44.2%) reporting the highest rates of probable PTSD. Those over 40 years and with lower educational attainment were also more likely to report PTSD symptoms.

### Mental health and blood pressure

The mean blood pressure was 130/79 mmHg (Table 2). Unadjusted models indicated that mean systolic blood pressure (SBP) was significantly lower in police employees who reported symptoms of depression or anxiety, whereas significantly raised levels of mean diastolic blood pressure (DBP) were found in those who reported depressive symptoms.

**Table 2:** Clinical features of police employees across probable mental disorders.

	All participants N = 40299	Probable depression (PHQ ≥10) Mean (SD)			Probable anxiety (HADSA ≥11) Mean (SD)			Probable PTSD (TSQ ≥6) Mean (SD)		
**Clinical features**		No	Yes	p-value	No	Yes	p-value	No	Yes	p-value
SBP (mmHg) mean (SD)	129.84 (15.0)	129.90 (15.0)	128.86 (14.9)	<0.001	129.97 (14.9)	127.96 (15.4)	<0.001	129.67 (15.0)	129.87 (14.8)	0.617
DBP (mmHg) mean (SD)	79.16 (10.0)	79.06 (10.0)	79.76 (10.4)	<0.001	79.14 (10.0)	79.10 (10.4)	0.816	79.06 (10.0)	79.96 (10.1)	<0.001
Pulse rate (bpm)	69.62 (11.3)	69.44 (11.3)	71.45 (11.4)	<0.001	69.45 (11.3)	71.60(11.3)	<0.001	69.52 (11.3)	70.21 (11.5)	0.021
Self-reported diagnosis of hypertension N (%)		N (%)	N (%)	<0.001	N (%)	N (%)	<0.001	N (%)	N (%)	0.083
No	37833 (94.0)	33603 (90.4)	3555 (9.6)		34080 (91.7)	3078 (8.3)		34104 (96.1)	1370 (3.9)	
Yes	2401 (6.0)	2056 (86.9)	309 (13.1)		2098 (88.7)	267 (11.3)		2162 (95.4)	104 (4.6)	

SBP, Systolic Blood Pressure; DBP, Diastolic Blood Pressure. HADSA, hospital anxiety and depression scale; PHQ, patient health questionnaire; PTSD, post-traumatic stress disorder; SD, standard deviation; TSQ, trauma screening questionnaire. Missing values were due to no assessment of those participant characteristics in a specific version of the questionnaire or no response received from participants. P-values have been derived from the Chi2 test for categorical variables and independent t-test for continuous variables.

No significant difference was found in the SBP among probable PTSD cases compared to non-PTSD cases, but DBP levels were significantly increased.

Six percent of police employees self-reported a diagnosis of hypertension and this percentage was significantly higher in those who reported symptoms of depression (13.1%) or anxiety (11.3%). No significant difference was found when looking at those reporting symptoms of PTSD (4.6%). However, when exploring this specifically among those who reported a traumatic exposure in the past six months and met the criteria for probable PTSD, 36.0% reported a hypertension diagnosis (S2 Table). Among those who reported symptoms of anxiety or depression, self-reported diagnosis of hypertension was 21.8% and 25.3%, respectively.

Adjusted models did not show a significant difference between mean SBP and probable anxiety, PTSD or depression caseness (Table 3). A different pattern was however found with DBP (Table 4); police employees who reported depressive symptoms had a significant higher mean DBP score, by 1.31 mmHg (95% CI 0.99, 1.64), than those who did not. Police employees meeting criteria for probable anxiety and PTSD were also found to have significantly increased mean levels of DBP than non-cases, 0.82 mmHg (95% CI 0.47–1.17) and 0.81 (95% CI 0.30–1.32), respectively.

**Table 3:** Associations between systolic blood pressure across probable mental disorders.

	Difference in mean SBP (mmHg)			
	Model 1	Model 2	Model 3	Model 4
	β (95% CI)	β (95% CI)	β (95% CI)	β (95% CI)
Symptoms of depression	**-1.04 (-1.54, - 0.54)**	**0.59 (0.15, 1.03)**	**0.57 (0.10, 1.03)**	0.46 (-0.01, 0.93)
Symptoms of anxiety	**-2.01 (-2.54, -1.48)**	0.33 (-0.15, 0.80)	0.40 (-0.11, 0.89)	0.23 (-0.27, 0.72)
Symptoms of PTSD	0.20 (-0.58, 0.98)	-0.08 (-0.77, 0.61)	0.27 (-0.46, 1.00)	0.10 (-0.63, 0.83)

CI, confidence interval; PTSD, post-traumatic stress disorder; SBP, Systolic Blood Pressure. Multivariate linear regression analyses were used.

Model 1: unadjusted model.

Model 2: adjusted model for gender and age (continuous).

Model 3: adjusted model for gender, age, educational attainment, marital status, ethnicity, job role.

Model 4: adjusted for gender, age, educational attainment, marital status, ethnicity, job role, BMI, exercise, smoking and alcohol consumption.

**Table 4:** Associations between diastolic blood pressure across probable mental disorders.

	Difference in mean DBP (mmHg)			
	Model 1	Model 2	Model 3	Model 4
	β (95% CI)	β (95% CI)	β (95% CI)	β (95% CI)
Symptoms of depression	**0.69 (0.36, 1.02)**	**1.45 (1.15, 1.76)**	**1.46 (1.13, 1.79)**	**1.31 (0.99, 1.64)**
Symptoms of anxiety	-0.04 (-0.40, 0.31)	**0.92 (0.59, 1.25)**	**0.99 (0.64, 1.34)**	**0.82 (0.47, 1.17)**
Symptoms of PTSD	**0.90 (0.37, 1.42)**	**0.75 (0.27, 1.24)**	**0.98 (0.47, 1.49)**	**0.81 (0.30, 1.32)**

CI, confidence interval; DBP, Diastolic Blood Pressure; PTSD, post-traumatic stress disorder. Multivariate linear regression analyses were used.

Model 1: unadjusted model.

Model 2: adjusted model for gender and age (continuous).

Model 3: adjusted model for gender, age, educational attainment, marital status, ethnicity, job role.

Model 4: adjusted for gender, age, educational attainment, marital status, ethnicity, job role, BMI, exercise, smoking and alcohol consumption.

## Discussion

This large, nationally representative, study identified that probable depression was the most prevalent disorder (9.8%) amongst police employees, followed by probable anxiety (8.5%), and then probable PTSD (3.9%). Police personnel most at risk of adverse mental health outcomes were police staff and those self-reporting trauma exposure in the past six months. For the latter, dependent on the mental health outcome of interest, a two to six-fold increase of the rate of probable mental disorders was found and this was strongly associated with heavy alcohol consumption. It was notable that a self-reported hypertension diagnosis was significantly more common among those with poorer mental health. However, after adjustment, only DBP remained positively associated with probable depression, anxiety or PTSD. Nevertheless, the differences were too small (≈1 mmHg) to have clinical significance.

### Prevalence of probable PTSD, depression and anxiety

We found substantially lower rates of probable mental disorders compared to other UK [5,40,41] and international studies [2,6,7,39,42] among police personnel. One of the largest and most recent surveys to date in the UK (2018), known as “The Job, The Life”, utilising data from 16,857 participants, reported that 90% of the police force experienced trauma and that 8% would go on to experience symptoms of PTSD and 12% symptoms of complex PTSD as measured with the international trauma questionnaire [40]. Moreover, the study found that amongst the 80% of individuals who did not experience PTSD, around half reported fatigue, anxiety, and trouble sleeping [40]. However, the survey focused on trauma management and working conditions among members of the police forces and this framing may explain the far higher rate of trauma exposure, and PTSD, compared to the current study which focused on the possible health impact of a newly introduced mobile radio system used by the UK police forces [13].

We consider the most likely explanation for the lower prevalence rates reported in the current study is the different methodology of the Airwave study. In particular, it is not just its size and representativeness: it is that most of the comparable studies, like ‘The Job, The Life’ and other studies focusing on first responders are described as occupational mental health or ‘stress’ surveys, and there is good evidence that high rates of adverse mental health outcomes are often found in studies that are framed as such [12,43]. Alternatively many studies have been carried out after a specific traumatic incident or disaster what may also result in elevated rates of adverse mental health outcomes [39]. However, a systematic review found that the prevalence of PTSD among police officers exposed to large-scale disasters or extreme events was in general lower than the prevalence found in the general population exposed to the same event [44]. In our study there was no framing around mental health and the study was not set up to explore the aftermath of a particular disaster. Instead, the study was specifically not a mental health study, but one in which the standard mental health measures were included but with little prominence. We suggest that the results of our, non-mental health focused, study are more likely to be a true representation of the mental health status of police employees.

The prevalence of probable mental disorders found among police employees in this study appears to be comparable to rates found in a systematic review exploring the prevalence of PTSD in different groups of emergency services personnel including the police [39]. This systematic review covered studies published between 1985 and 2007 and found that only 5% of police officers exposed to a major disaster presented with PTSD. This rate was the lowest compared to the other groups of rescue personnel (e.g. ambulance personnel, firefighters and other rescue teams) investigated [39]. These levels of PTSD are comparable to those found amongst UK military personnel, with rates of probable PTSD and CMD around 6% and 22%, respectively [45]. Research conducted among ambulance staff in the UK reported higher levels of PTSD and anxiety (both at 22%) [46]. A systematic review and meta-analysis including data from over 30,000 ambulance personnel worldwide found a PTSD prevalence rate of 11%, with CMD being more prevalent at 15% [47]. Possible explanations for higher rates of mental disorders among ambulance staff compared to other first responders’ populations, may include a higher occupationally-related trauma-burden such as closer proximity to the suffering of others and hence increased feelings of guilt if they fail to provide the assistance needed [39,48]. In addition, police personnel are subject to stringent employment selection procedures to join the police force and this may lead to a more resilient workforce [2].

The overall rates of probable CMD and PTSD among UK police officers in the current study were in fact comparable to the rates in the UK general population (17% and 4%, respectively) [49]. However, the findings may be affected by the ‘healthy-worker’ effect wherein working individuals are physically and mentally healthier than non-working individuals [50]; our study only included active police officers and staff, whereas employees who were unable to cope with the stressors of police work may have left. Nevertheless, police employees are more frequently exposed to traumatic events in the line of duty compared to people working in other occupations, suggesting that most police employees who remain in the police force are an increasing resilient group of workers.

### Groups at risk for probable PTSD, depression and anxiety

The findings reveal that among police officers and staff who reported exposure to a traumatic event in the past 6 months, 27% met criteria for probable PTSD. In addition, symptoms of CMD were about double among this trauma exposed group compared to those who were not similarly exposed. Given the overall rates of probable mental disorders were similar to those in the general population, it follows that either most police employees recover over time or leave service. However, given that mental ill-health adversely impacts on occupational functioning [51], often referred to as presenteeism, our findings suggest that police forces should pay particular attention to recently trauma-exposed staff to ensure that they are able to safely carry out the roles they undertake, especially for safety critical staff such as firearms officers. A caveat that should be considered is the cross-sectional nature of the data. We were unable to establish whether there was a causal relationship between the traumatic event and the subsequent development of PTSD or whether the PTSD identified was related to the traumatic event the participant reported to have experienced within the last 6 months. For example, personnel may have had a history of psychiatric illness. Furthermore, recall bias may have influenced our findings as it is well evidenced that the reporting of traumatic events may be increased among people with current mental disorders [52]. This is partially suggested by the fact that the association with traumatic events was not specific to PTSD, although many studies are showing that post-trauma ill health is not just restricted to PTSD [53]. This should be explored in detail once the follow-up data collection for the Airwave Study has been finalised.

A trend was seen across all three mental health outcomes, whereby poorer mental health was consistently associated with drinking alcohol more heavily. This was especially of concern among the police officers who had experienced trauma in the last six months and met the criteria for PTSD, of whom 42% reported levels of alcohol consumption defined as increasing or high risk to health. Previous studies confirm these findings and showed that heavy alcohol consumption was associated with depression, anxiety, and PTSD [54–56]. Addressing drinking that is likely to be harmful to health should be a priority as in the long run this may also impair the physical health of police employees and lead to possible occupational safety risks. Of note, this study did not examine whether those with poor mental health, or those drinking at harmful levels, were seeking professional help; this should be a focus of future studies given the impact of presenteeism on the ability of police employees to effectively protect the public.

Other groups at risk in our sample included lower ranking personnel, women, those not in a relationship and with poorer lifestyle habits (i.e. smoking, less physical exercise). Across population surveys and surveys focused on particular first responder occupations similar factors have found to be associated with an increased risk of suffering poor mental health [5,8,39,49].

### Mental disorders, hypertension and blood pressure

This study revealed that self-reported hypertension was significantly higher in those who reported these adverse mental health outcomes. Previous findings have showed mixed results, with some suggesting that depression and anxiety were not associated with hypertension [21], and others finding a positive association between depression and hypertension [22]. However, after adjustments, whilst the mean levels of DBP remained significantly higher across all three mental health outcomes, this difference was minimal and not likely to be of clinical significance. Higher DBP levels were also found in a meta-analysis exploring differences in blood pressure among people with PTSD, compared to two control samples, namely a trauma non-PTSD group and a non-trauma group. For SBP, levels were only significantly elevated when comparing those with PTSD to the non-trauma control group. Still as in the current study, blood pressure deviations were only small (range 1–5 mmHg) [57]. In the current study, unfortunately, no comprehensive data were available to us on the uptake of anti-hypertension medication, a possible confounder. However, the associations found were similar between SBP, DBP and adverse mental health outcomes after adjustment for self-reported hypertension diagnosis in further analyses. We also re-ran the analyses without those who reported a hypertension diagnosis and similar findings were found.

### Strengths and limitations

The strength of this study is that it uses data from the largest cohort study of police employees that currently exists worldwide. The fact that the study was set up to examine the impact of a new communication system on the health of UK police forces employees is another strength of the current study, because this focus on physical health instead of mental health will have limited the impact of a framing effect. Still, there are limitations. The mental health measures do not provide a clinical diagnosis as they are based on self-report and not a clinical interview, however the measures used have shown to be valid and reliable [30–33]. Data were collected by advertising access to a full, free health screen. This may have introduced selection bias as concerned individuals and those who are particularly interested in staying healthy may have been more likely to participate. Moreover, police employees who are less able to cope with the stress of the job may have left the police. Further, no data were available to us regarding how long respondents had been part of the UK police force. It was not possible to look at police constables and sergeants independently, who differ greatly in terms of job role, due to questionnaire design. Unfortunately, blood pressure was only measured in a single setting with a sphygmomanometer. Ambulatory blood pressure monitoring would have been preferred for more accurate assessment but was not feasible in the current study due to the large sample size. Finally, no data were available to us regarding factors that previously have found to be associated with the mental disorders and blood pressure such as shift work, sleep and type of trauma exposure [2,3,20,58]. Additional research is needed to explore this in further detail among members of the police force in Great Britain.

### Implications

Our data suggest that the overall rates of poor mental health among police officers and staff were not substantially different than those of the general population. However, respondents who had been recently exposed to traumatic situations had elevated rates of probable PTSD, CMD and heavy alcohol use. Our results suggest that this group, likely to be working in response roles, may require additional mental health support and encouragement to seek professional help in order to minimise the impact of presenteeism. This is especially important for police employees in safety critical roles. The current NICE guidelines recommend a period of active monitoring for evidence of clinically important PTSD symptoms and such an approach may be helpful for those regularly exposed to trauma [59].

Another important finding is that, overall, lower ranking staff (police staff/constables/sergeants) reported poorer mental health than higher rank staff (inspector and above). This suggests that mental health initiatives put in place within the police need to be focused across all employees and not just those in uniform. This is especially important as poor mental health may lead to functional impairment [51,60,61] and affects the ability of members of emergency services personnel, including the police, to protect the public during stressful and traumatic situations [8]. Furthermore, despite prior research focusing mainly on PTSD among police personnel, the higher prevalence of CMD among police officers and staff indicate that prevention, early identification and treatment initiatives should focus on the complete spectrum of mental disorders, and not solely on PTSD.

Unhealthy lifestyle habits such as smoking, limited exercise and heavy drinking were associated with probable mental disorders in the current study. As identified from previous research, these factors also increase the risk of hypertension [20]. Therefore, we suggest that it is important that police employees continue to be encouraged to have healthy lifestyles. One focus should be on alcohol consumption, as high alcohol consumption was associated with mental illness amongst police. However, we suggest that interventions to improve officer’s use of alcohol need to be evidence based and reliance on alcohol education, which has not been found to be effective, is unlikely to be helpful in isolation [62]. Ensuring that alcohol use policies are adhered to and individuals who do drink heavily are helped in a supportive and non-stigmatised manner may be more useful.

It is well-evidenced from research among military personnel and the first responder population that stigma and barriers to care may hamper or delay help-seeking for mental health problems [63–65]. The most common stigma related concerns include worries about career repercussions and confidentiality. Difficulties in arranging an appointment and not knowing where to go for help were most frequently mentioned when exploring barriers to mental health care. These concerns need to be considered when developing strategies to encourage help-seeking for mental health problems for police employees, especially as research has shown that these concerns are most prevalent among those with the most severe mental health symptoms [60,63,66].

## Conclusions

Using the Airwave Health Monitoring Study, this study provided a comprehensive overview of the mental health of UK police employees, possible risk factors and associations with blood pressure. The majority of the UK police force appear to have good mental health, however we identified that recently trauma-exposed employees, police staff and heavy drinkers are particularly likely to report poor mental health. Despite our finding that there was a significant link between DBP and mental disorders, this association was unlikely to be clinically relevant. These findings highlight the importance of continued health monitoring of members of the UK police force, with a focus on police staff and those with recent trauma exposure.

## Supporting information

S1 Table Characteristics and probable mental disorders among police employees who reported a traumatic exposure in the past 6 months. (DOCX)

S2 Table Clinical features and probable mental disorders among police employees who reported a traumatic exposure in the past 6 months (n = 5,454). (DOCX)
