# Supplementary material for: Probable PTSD, depression and anxiety in 40,299 UK police officers and staff: Prevalence, risk factors and associations with blood pressure
Source: PLoS One. 2020 Nov 12;15(11):e0240902. doi: 10.1371/journal.pone.0240902 (PMC7660485; doi:10.1371/journal.pone.0240902)
Supplement: S1 Table — (DOCX) [file pone.0240902.s001.docx]

Supplementary Table 1: Characteristics and probable mental disorders among police employees who reported a traumatic exposure in the past 6 months.

| Characteristics | Probable depression  (PHQ ≥10) | | | Probable anxiety  (HADS ≥11) | | | Probable PTSD  (TSQ ≥6) | | |
| --- | --- | --- | --- | --- | --- | --- | --- | --- | --- |
|  | No | Yes | p-value | No | Yes | p-value | No | Yes | p-value |
| **Overall** n (%) | 4481 (82.2) | 972 (17.8) |  | 4561 (83.6) | 892 (16.4) |  | 3980 (73.0) | 1474 (27.0) |  |
| **Gender** n (%) |  |  | <0.001 |  |  | <0.001 |  |  | 0.324 |
| Female | 1589 (79.3) | 414 (20.7) |  | 1580 (78.8) | 423 (21.1) |  | 1478 (73.8) | 526 (26.3) |  |
| Male | 2892 (83.8) | 558 (16.2) |  | 2981 (86.4) | 469 (13.6) |  | 2502 (72.5) | 948 (27.5) |  |
| **Age** n (%) |  |  | 0.462 |  |  | 0.096 |  |  | <0.001 |
| < 30 years | 668 (82.5) | 142 (17.5) |  | 691 (85.3) | 119 (14.7) |  | 647 (79.9) | 163 (20.1) |  |
| 30-39 years | 1602 (82.3) | 345 (17.7) |  | 1644 (84.4) | 303 (15.6) |  | 1487 (76.4) | 460 (23.6) |  |
| 40-49 years | 1779 (82.6) | 375 (17.4) |  | 1788 (83.0) | 366 (17.0) |  | 1487 (69.0) | 668 (31.0) |  |
| > 49 years | 432 (79.7) | 110 (20.3) |  | 438 (80.8) | 104 (19.2) |  | 359 (66.2) | 183 (33.8) |  |
| **Marital status** n (%) |  |  | <0.001 |  |  | 0.002 |  |  | 0.164 |
| Married/cohabiting | 3514 (83.7) | 687 (16.4) |  | 3546 (84.4) | 655 (15.6) |  | 3076 (73.2) | 1126 (26.8) |  |
| Single | 548 (79.8) | 139 (20.2) |  | 574 (83.6) | 113 (16.5) |  | 514 (74.8) | 173 (25.2) |  |
| Divorced/separated | 304 (73.6) | 109 (26.4) |  | 323 (78.2) | 90 (21.8) |  | 286 (69.3) | 127 (30.8) |  |
| Other | 109 (75.7) | 35 (24.3) |  | 111 (77.1) | 33 (22.9) |  | 100 (69.4) | 44 (30.6) |  |
| **Ethnicity** n (%) |  |  | 0.255 |  |  | 0.790 |  |  | 0.179 |
| White | 4241 (82.4) | 908 (17.6) |  | 4308 (83.7) | 841 (16.3) |  | 3770 (73.2) | 1380 (26.8) |  |
| Other | 228 (79.7) | 58 (20.3) |  | 241 (84.3) | 45 (15.7) |  | 199 (69.6) | 87 (30.4) |  |
| **Education** n (%) |  |  | 0.030 |  |  | 0.632 |  |  | 0.011 |
| Vocational qualifications | 312 (78.8) | 84 (21.2) |  | 337 (85.1) | 59 (14.9) |  | 281 (70.8) | 116 (29.2) |  |
| GCSE equivalent or below | 1339 (80.8) | 319 (19.2) |  | 1373 (82.8) | 285 (17.2) |  | 1172 (70.7) | 486 (29.3) |  |
| A levels / higher or equivalent | 1472 (82.7) | 307 (17.3) |  | 1488 (83.6) | 291 (16.4) |  | 1304 (73.3) | 475 (26.7) |  |
| Bachelor / postgraduate degree | 1352 (83.9) | 260 (16.1) |  | 1356 (84.1) | 256 (15.9) |  | 1219 (75.6) | 393 (24.4) |  |
| **Role** n (%) |  |  | <0.001 |  |  | <0.001 |  |  | 0.007 |
| Police staff | 703 (77.4) | 205 (22.6) |  | 710 (78.2) | 198 (21.8) |  | 620 (68.2) | 289 (31.8) |  |
| Police constable/sergeant | 2870 (82.1) | 624 (17.9) |  | 2947 (84.3) | 547 (15.7) |  | 2563 (73.4) | 931 (26.7) |  |
| Inspector or above | 379 (88.8) | 48 (11.2) |  | 368 (86.2) | 59 (13.8) |  | 315 (73.8) | 112 (26.2) |  |
| **Takes intensive exercise** n (%) |  |  | <0.001 |  |  | 0.037 |  |  | 0.436 |
| No | 3907 (81.6) | 884 (18.5) |  | 3990 (83.3) | 801 (16.7) |  | 3488 (72.8) | 1304 (27.2) |  |
| Yes | 566 (86.8) | 86 (13.2) |  | 564 (86.5) | 88 (13.5) |  | 484 (74.2) | 168 (25.8) |  |
| **Currently smoking** n (%) |  |  | <0.001 |  |  | 0.086 |  |  | 0.499 |
| No | 4092 (82.9) | 844 (17.1) |  | 4142 (83.9) | 794 (16.1) |  | 3608 (73.1) | 1328 (26.9) |  |
| Yes | 387 (75.2) | 128 (24.9) |  | 417 (81.0) | 98 (19.0) |  | 370 (71.7) | 146 (28.3) |  |
| **Alcohol consumption#**  n (%) |  |  | <0.001 |  |  | <0.001 |  |  | <0.001 |
| Non-drinker | 399 (75.9) | 127 (24.1) |  | 419 (79.7) | 107 (20.3) |  | 378 (71.7) | 149 (28.3) |  |
| Low risk | 2446 (83.8) | 474 (16.2) |  | 2491 (85.3) | 429 (14.7) |  | 2214 (75.8) | 706 (24.2) |  |
| Increasing risk | 1509 (83.5) | 299 (16.5) |  | 1511 (83.6) | 297 (16.4) |  | 1270 (70.2) | 538 (29.8) |  |
| High risk | 111 (61.3) | 70 (38.7) |  | 123 (68.0) | 58 (32.0) |  | 101 (55.8) | 80 (44.2) |  |
| **BMI** (kg/m2) | 27.28 (4.18) | 28.74 (4.85) | <0.001 | 27.45 (4.27) | 27.99 (4.66) | <0.001 | 27.28 (4.20) | 28.24 (4.63) | <0.001 |

BMI, body mass index; GCSE, General Certificate of Secondary Education; HADSA, hospital anxiety and depression scale; PHQ, patient health questionnaire; PTSD, post-traumatic stress disorder; SD, standard deviation. ^#^Alcohol drinking was based on alcohol units calculated from different type of drinks/beverages consumed in the past week using sex-specific cut offs: non-drinker, not drinking alcohol; low risk < 14 units men and women; increasing risk 14-35 units for women, 14-50 units for men; high risk >35 units women and >50 units for men. BMI was missing if a participant did not attend the health screening. Other missing values were due to no assessment of those participant characteristics in a specific version of the questionnaire or no response received from participants. P-values have been derived from the Chi2 test for categorical variables and independent t-test for continuous variables.
