# Supplementary material for: Probable PTSD, depression and anxiety in 40,299 UK police officers and staff: Prevalence, risk factors and associations with blood pressure
Source: PLoS One. 2020 Nov 12;15(11):e0240902. doi: 10.1371/journal.pone.0240902 (PMC7660485; doi:10.1371/journal.pone.0240902)
Supplement: S2 Table — (DOCX) [file pone.0240902.s002.docx]

Supplementary Table 2: Clinical features and probable mental disorders among police employees who reported a traumatic exposure in the past 6 months (n=5,454).

|  | Probable depression (PHQ ≥10)  Mean (SD) | | | Probable anxiety (HADSA ≥11)  Mean (SD) | | | Probable PTSD (TSQ ≥6)  Mean (SD) | | |
| --- | --- | --- | --- | --- | --- | --- | --- | --- | --- |
| **Clinical features** | No | Yes | p-value | No | Yes | p-value | No | Yes | p-value |
| SBP (mmHg) | 129.24 (14.73) | 129.47 (15.11) | 0.649 | 129.48 (14.66) | 128.27 (15.46) | 0.027 | 129.05 (14.80) | 129.87 (14.79) | 0.069 |
| DBP (mmHg) | 78.91 (9.92) | 80.18 (10.35) | <0.001 | 79.09 (9.95) | 79.39 (10.30) | 0.399 | 78.83 (9.98) | 79.96 (10.05) | <0.001 |
| Pulse rate (bpm) | 69.11 (11.11) | 71.47 (11.54) | <0.001 | 69.22 (11.20) | 71.12 (11.20) | <0.001 | 69.27 (11.10) | 70.21 (11.51) | 0.061 |
| Diagnosis of hypertension | N (%) | N (%) | 0.001 | N (%) | N (%) | 0.010 | N (%) | N (%) | 0.001 |
| No | 4262 (82.6) | 899 (17.4) |  | 4332 (83.9) | 829 (16.1) |  | 3792 (73.5) | 1370 (26.5) |  |
| Yes | 216 (74.7) | 73 (25.3) |  | 226 (78.2) | 63 (21.8) |  | 185 (64.0) | 104 (36.0) |  |

SBP, Systolic Blood Pressure; DBP, Diastolic Blood Pressure.
